# The Contemporary Role of Oral Maxillofacial Surgeons in Head and Neck Reconstructions

**DOI:** 10.3390/jcm15103928

**Published:** 2026-05-20

**Authors:** Hisham Marwan, Camilo Mosquera, Srinivasa R. Chandra

**Affiliations:** 1 Department of Surgery, The University of Texas Medical Branch, Galveston, TX 77555, USA; camosque@utmb.edu; 2 Swedish Providence Hospital, Seattle, WA 98122, USA; 3 Veterans Affairs, Portland, OR 97239, USA; 4 Madigan Army Medical Center, Joint Base Lewis-McChord, Tacoma, WA 98431, USA

**Keywords:** oral maxillofacial surgery, microsurgery, oral head and neck neoplasms, free tissue flaps, treatment outcome, multidisciplinary care team

## Abstract

Oral and Maxillofacial Surgery (OMFS) is a surgical specialty with a distinctive position at the intersection of medicine and dentistry. This unique expertise enables reliable reconstructions of complex head and neck defects, with a focus on function, esthetics, and quality of life. This review examines the historical progression, current practices, and prospective directions of head and neck reconstruction, with particular emphasis on the essential contributions of Oral and Maxillofacial Surgeons (OMSs). Beginning with early reconstructive efforts in ancient civilizations and progressing through the transformative advancements of the Renaissance, the introduction of anesthesia and antiseptics, and innovations during periods of war, the specialty has evolved in response to increasing clinical complexity. The contemporary era is characterized by the integration of microvascular reconstruction, dental rehabilitation, and advanced imaging modalities, enhancing the restoration of occlusal function, facial aesthetics, and overall quality of life. Emerging innovations such as patient-specific three-dimensional printed hardware, tissue engineering, regenerative medicine, artificial intelligence, and supermicrosurgical techniques are expected to further reshape reconstructive approaches. These technological advances aim to reduce the number of surgical steps, improve biological reconstruction, and enhance diagnostic and planning capabilities. However, they also raise ethical considerations and validation challenges that warrant careful assessment. In conclusion, Oral and Maxillofacial Surgeons continue to play a central and expanding role in head and neck reconstruction and rehabilitation. Owing to comprehensive training and technological expertise, the specialty is uniquely positioned to advance value-based, multidisciplinary care while persistently striving to improve functional, aesthetic, and quality-of-life outcomes for patients with complex craniofacial defects.

## 1. Introduction

Oral and Maxillofacial Surgery (OMFS) is the bridging specialty between dentistry and medicine. Oral and Maxillofacial Surgeons (OMSs) are integral to both dental and surgical teams. The OMFS specialty encompasses a diverse range of practices, including wisdom tooth extractions, dental implants, cranio-maxillofacial (CMF) trauma, orthognathic surgery, obstructive sleep apnea, craniofacial surgery, skull base surgery, head and neck cancer, and microvascular reconstruction. In the United States, OMSs receive additional anesthesia training to allow them safely administer anesthesia in their practices.

The breadth of this specialty stems from the comprehensive training and extensive knowledge base required for board certification. Certification may be obtained through regional boards, such as the American Board of Oral and Maxillofacial Surgery (ABOMS), or through international organizations, including the International Board for Certification of Specialists in Oral and Maxillofacial Surgery (IBCSOMS). The IBCSOMS is an international board that aims to identify a core curriculum and training for an oral and maxillofacial surgery training program worldwide and to establish a certification process to demonstrate that a candidate has met that core level of training. Furthermore, the specialty now offers fellowship training in subspecialties such as Head and Neck Oncology, Microvascular Reconstruction, Cleft and Craniofacial Surgery, and Cosmetic Surgery. Each subspecialty has its own board certification or certificate of added qualification.

The field of head and neck reconstruction has evolved through a multidisciplinary approach, with significant contributions from general surgery, Oral and Maxillofacial surgery, Plastics Surgery, and Otolaryngology. Each specialty offers distinct expertise, collectively advancing techniques and patient care. Currently, optimal patient outcomes and quality of life are achieved through collaborative multidisciplinary teams, reflecting the balanced integration of these specialties. Because of extensive training in both medicine and dentistry, the OMS is exceptionally qualified as a reconstructive surgeon capable of precisely restoring function, esthetics, and quality of life for patients with head and neck defects. The OMS’s ability to combine surgical and dental expertise enables patients to receive comprehensive reconstructive care in a single setting, helping them regain a normal quality of life and restore function and esthetics.

This review aims to provide a comprehensive overview of the historical evolution of maxillofacial reconstruction. Emphasis will be placed on the pivotal role of Oral and Maxillofacial Surgery in advancing the field of head and neck reconstruction, as well as the breadth of procedures commonly performed in contemporary practice. We will examine current reconstructive techniques, evaluate their limitations, and highlight prospective directions for innovation and growth within the specialty.

## 2. History

Throughout history, the passage of time inevitably leads to the loss or erasure of certain events, individuals, and knowledge. In this overview, we will provide a comprehensive account of the history of Oral and Maxillofacial surgery, tracing its origins from ancient civilizations such as India and Egypt, through significant developments in the Middle Ages and Renaissance, and culminating in the advanced techniques and innovations of the modern era. By examining pivotal milestones, influential figures, and groundbreaking procedures, we aim to describe the specialty’s dynamic evolution and highlight its impact on patient care and surgical practice.

Since antiquity, surgeons have endeavored to restore facial structures. As early as 1600 BC, Egyptians authored the Edwin Smith Papyrus—acquired by its namesake in 1862—which is regarded as the oldest known surgical treatise on trauma, including maxillofacial fractures [1]. Around 600 BC, Sushruta, the earliest known surgeon to use local tissue flaps for nasal reconstruction, is widely regarded as the father of plastic surgery [2,3].

Between the 5th century BC and the 1st century AD, Greek contributions to medicine and surgery were preeminent. Hippocrates established the dictum, “First, do no harm,” focusing primarily on fracture management while devoting less attention to reconstructive techniques [4].

He is credited with first describing the terms “carcinoma” and “carcinos” in relation to tumor growth [5,6]. The Romans further developed Greek medical understanding. The reconstructive work of Aulus Cornelius Celsus, particularly his repairs of the lip and ear, served as precursors to modern reconstructive surgery [7].

During the Islamic Golden Age, prior medical knowledge was preserved and significantly advanced, with an emphasis on causal reasoning in surgical practice. Operations were performed in Bimaristans (specialized hospitals) using innovative surgical techniques and new instruments [8]. Al-Zahrawi (Albucasis) authored ‘Al-Tasrif’ (The Method of Medicine), a seminal surgical compendium translated into multiple languages, and he was the first to distinguish oral surgery as a separate discipline [8].

During the Renaissance, Andreas Vesalius published his seminal work, ‘De Humani Corporis Fabrica, in 1543, offering detailed anatomical descriptions of the human body, including the jaws and facial skeleton [8]. Concurrently, amid the spread of syphilis, the Italian surgeon Gaspare Tagliacozzi pioneered the first nasal reconstruction technique using tissue harvested from the arm [9].

Two seminal breakthroughs transformed all surgical specialties: the introduction of anesthesia and the development of antiseptic techniques. In 1846, William T.G. Morton successfully anesthetized Edward Gilbert Abbott for a tumor excision under the supervision of surgeon John Collins Warren at Massachusetts General Hospital’s Ether Dome [10]. This demonstration provided evidence of pain eradication, leading to widespread adoption. In the 1860s, Joseph Lister established the principles of antisepsis, demonstrating that bacteria were responsible for fermentation, wound infection, and sepsis. These advancements enabled surgeons to undertake complex operations with dramatically reduced mortality, laying the foundation for the emergence of surgical specialties [11].

Among the factors contributing to the growth of Oral and Maxillofacial Surgery, the treatment of war-related injuries stands paramount. The advent of gunpowder, cannonballs, and musket balls in the sixteenth century produced unprecedented facial trauma and tissue loss. This trend intensified during World Wars I and II, when the specialty advanced rapidly in response to the extensive facial injuries sustained by soldiers. During World War I, Harold Gillies and Varaztad Kazanjian managed over 5000 patients with complex facial defects, pioneering techniques such as tube flaps and bone-graft reconstructions [12,13]. Kazanjian subsequently recorded their collaborative work in his 1946 publication, ‘The Surgical Treatment of Facial Injuries,’ and is widely regarded as the father of modern Oral and Maxillofacial Surgery.

## 3. Contemporary Oral and Maxillofacial Surgery

Oral and maxillofacial surgeons (OMSs) are an integral part of the multidisciplinary team and primary providers of facial trauma care, oral cancer treatment, and complex head and neck reconstruction. The breadth of training in these subspecialties and head-and-neck subsites adds to OMSs’ expertise in delivering care [14].

- Oral, Head & Neck Oncology and Reconstruction:

Oral and Maxillofacial Surgery is a pivotal component of the comprehensive management of oral cancer worldwide. OMS specialists are essential not only in the reconstruction following resections, but also in the management of patients receiving chemotherapy, targeted immunotherapy, and in addressing complications arising from radiation therapy. These clinical interventions are standard practice within OMFS centers worldwide. The National Comprehensive Cancer Network (NCCN) Head and Neck Cancer Guidelines formally recognize OMFS as a fundamental member of the multidisciplinary oncologic care team. Additionally, the guidelines emphasize that all patients should be assessed by a head and neck surgical oncologist prior to the commencement of therapy, with OMS specialists making contributions to surgical planning, reconstructive procedures, and dental rehabilitation [15]. In Germany, 75% of OMFS departments hold certification as head and neck cancer centers, with 97.83% presenting cases at multidisciplinary tumor boards and 97.73% prioritizing critical osseous reconstruction post-tumor resection. OMFS departments in Germany demonstrate a high degree of standardization in oncologic care, with 82.4% reporting improvements attributed to interdisciplinary collaboration (63.64%) and structural advancements (61.76%) [16].

## 4. Recent Advancements in Clinical Practice for Head and Neck Reconstruction

With advances in technology and diagnostic imaging, Oral and Maxillofacial surgeons have been at the forefront, incorporating these innovations and applying their dental training to fully restore function and quality of life for patients who have undergone cancer surgery or have severe facial defects.

In 1989, Hidalgo was the first to describe the use of the microvascular fibula flap for mandibular reconstruction [17]. With improvements in microvascular techniques and the development of titanium reconstruction plates, the microvascular fibula flap is considered the gold standard for mandibular reconstruction. In parallel, the field of Oral and Maxillofacial Surgery was using dental implants to restore missing dentition, and attempts were made to place dental implants in the fibula to support future dental rehabilitation following jaw reconstructions [18].

**Figure 1:**
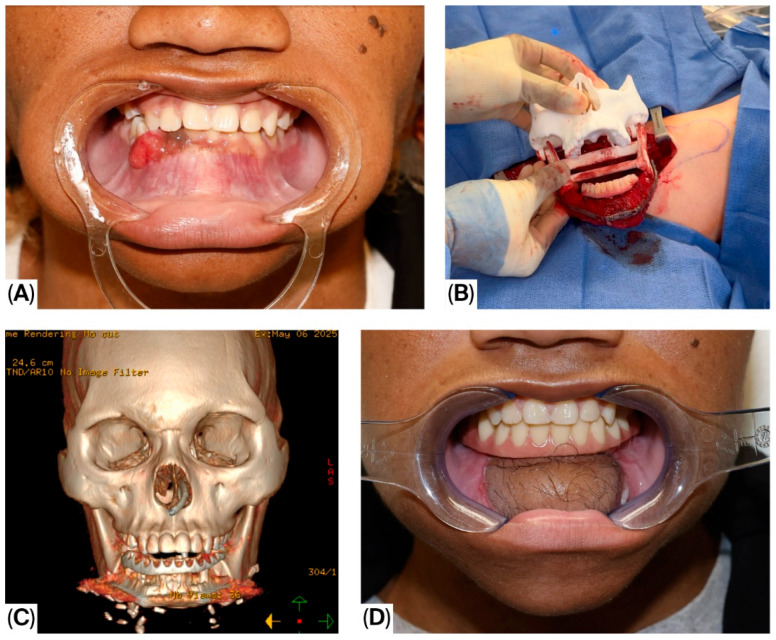
Jaw In A Day surgery. (**A**) A 25-year-old male presented with an anterior mandibular ameloblastoma. (**B**) Utilize the Virtual Surgical Plan (VSP) and cutting guides to adapt the three segments of fibula bone into the 3D-printed model. This approach allows for verification of occlusion and ensures proper flap orientation. (**C**) Postop CT scans and 3D reformatting. (**D**) 2 months postop.

With advances in imaging and the introduction of virtual surgical planning (VSP), reconstruction accuracy has improved, reducing operating time and improving patient outcomes. Oral and Maxillofacial Surgeons have incorporated the concept of ‘backward planning’ or functional rehabilitation-oriented reconstruction to provide patients with immediate dental implantation and prosthetic rehabilitation in a procedure known as “Jaw In A Day” (JIAD) in Figure 1 [18,19].

**Figure 2:**
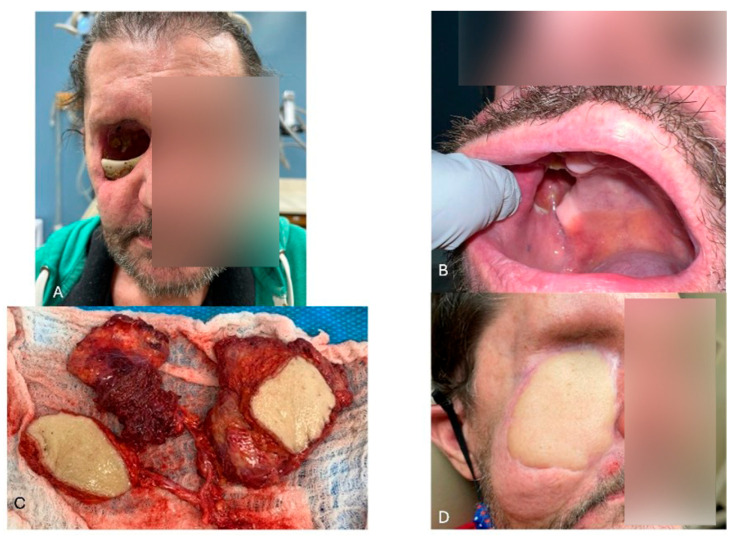
Complex midface reconstruction after trauma. (**A**) 60-year-old male patient presented with a large maxillectomy defect after a self-inflicted injury. (**B**) Note the intraoral communication. (**C**) Harvest of chimeric scapula and latissimus dorsi muscle flap to reconstruct the multiple components of the defect. (**D**) 3-month follow-up.

**Figure 3:**
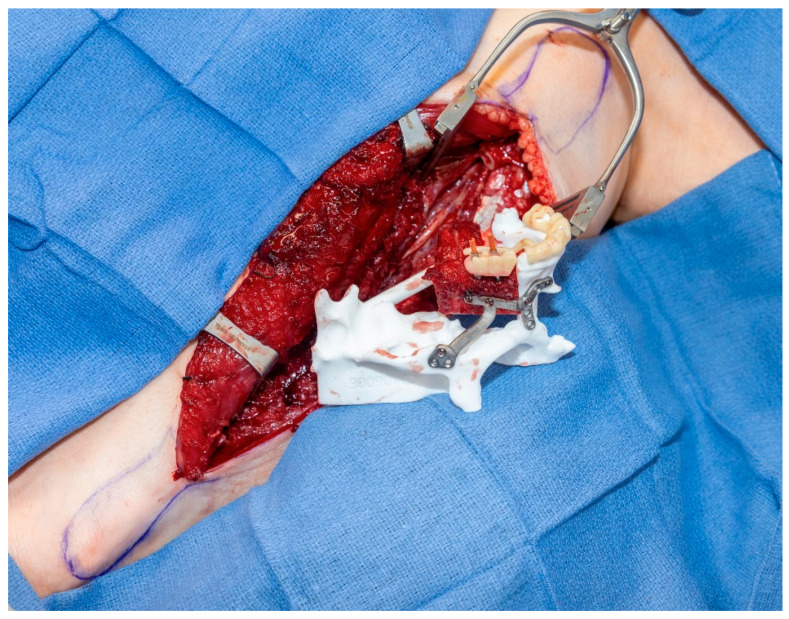
A case demonstrating the use of the JIAD procedure for maxillary reconstruction.

As with the mandible, innovations in midface reconstruction have markedly improved both functional and esthetic outcomes. OMSs’ remain the primary specialists responsible for these complex reconstructions, evolving from traditional obturators to contemporary techniques that are functionally and cosmetically superior. Maxillary reconstruction using microvascular free flaps, such as those harvested from the fibula, iliac crest, or scapula, has become a gold standard. (Figure 2) Additionally, the “Jaw in a Day” procedure offers outstanding results for maxillary defects, enabling immediate dental rehabilitation. Meticulous planning of the vertical dimension of occlusion is critical, allowing precise restoration of the maxillary arch in seamless harmony with the patient’s dentition in Figure 3 [20,21].

**Figure 4:**
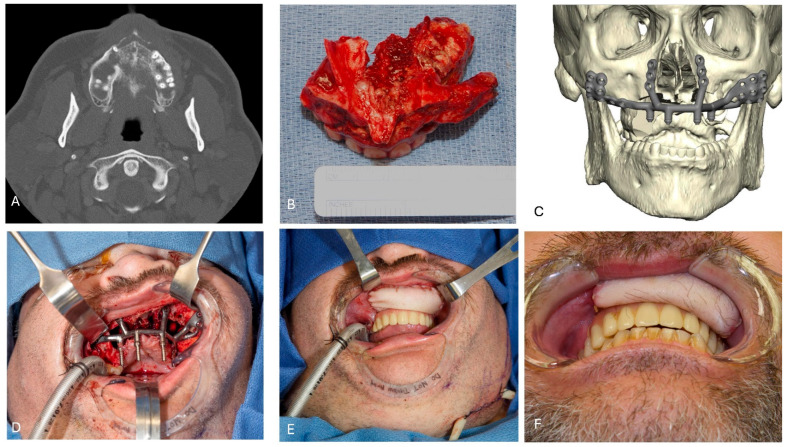
The use of IPS with a microvascular flap for maxillary reconstruction. (**A**) 44-year-old male with a secondary chronic maxillary osteomyelitis due to aspergillosis. (**B**) Infra-structure maxillectomy specimen. (**C**) Virtual surgical plan (VSP) for the IPS implant. (**D**) Intraoperative clinical picture showing the fixation of the IPS implant to restore the missing bone and dental structures. (**E**) Inset of the radial forearm free flap to close the oro-nasal and oro-antral communication. (**F**) 3 months follow-up.

Recent advances in the field have empowered surgeons to employ less invasive techniques while fully leveraging emerging technologies. The advent of three-dimensional (3D) printing, along with advances in biomaterials and biomechanics, enables the use of patient-specific, 3D-printed hardware for precise bone reconstruction. Furthermore, these innovations facilitate the simultaneous integration of dental components, allowing for immediate delivery of prosthetic solutions and significantly improving patient outcomes in Figure 4.

## 5. Future Directions

### 5.1. Tissue Engineering and Regenerative Medicine

As knowledge in tissue engineering advances, the ultimate goal is to restore missing tissues using allogenic substances that promote tissue regeneration. Numerous studies have explored replacing cartilage and bone tissues in the human body, particularly within the maxillofacial region. Currently, the standard approach for oral and maxillofacial reconstruction combines cadaveric bone grafts with bone morphogenetic protein (rh-BMP-2) and bone marrow aspirate concentrate (BMAC). This composite is stabilized with a titanium mesh or crib. The success of this method relies on the presence of healthy soft tissue surrounding the graft and adequate separation between the oral cavity and the grafted mixture [22].

Future directions will focus on establishing the appropriate proportions for the BMP, BMAC, and allogenic bone graft mixture. Additionally, advances in scaffold technology and the bioprinting of biological scaffolds are expected to enhance patient outcomes by reducing the number of required surgeries and improving the quality of regenerated bone [23].

### 5.2. Artificial Intelligence

The transformative impact of artificial intelligence (AI)/ machine learning (ML) is imminent. The integration of AI into OMFS has already resulted in significant advances, such as improved risk stratification for nerve injury during third molar extraction and dental implant procedures [24]. Additionally, AI can now perform automated cephalometric analyses and assist with presurgical orthognathic planning [25]. Ongoing research focuses on leveraging AI for the detection of premalignant lesions, early diagnosis of oral cancer, and assessment of lymph node involvement, with the expectation that these applications will be incorporated into routine clinical practice in the near future [26].

Recent studies have shown that supervised data entry to the AI/M could help detect the presence or absence of cervical metastasis. Using tubular data to train the model to predict the outcome variable of interest is feasible and can be highly effective for disease prediction. Similarly, the AI/ML model can be trained to predict continuous variables such as Disease-Free Survival (DFS) and overall survival (OS) rates [27,28].

Alongside these technological advancements, several ethical considerations must be addressed, including ensuring informed consent, protecting data privacy, and mitigating potential biases and equity issues related to data quality [29]. In addition, with any AI/ ML, the ability to broadly apply the model depends on the data entered. Therefore, rigorous validation and comprehensive evaluation are essential prerequisites for widespread implementation, ensuring that AI-driven outcomes are reliable, reproducible, and applicable to the broader patient population [30]. The specialty should allocate resources and plan strategically to ensure that it will be the leader in AI use, leveraging this technology to advance patients’ care and the specialty alike. Since this technology relies solely on a high-quality, large database, collaboration with other head and neck societies is prudent to help access and build such a database to develop clinical applications that directly impact patient care [31].

### 5.3. Supermicro Surgery and Lymphedema Surgery

Lymphedema of the head and neck is not uncommon and poorly understood. It is underdiagnosed, and the current treatment with lymphedema therapy is unpredictable [31]. Lymphaticovenous anastomosis (LVA) has been successful in the treatment of lymphedema of the lower limb. Fewer studies report a similar outcome in the head and neck region. Collaboration with the lymphedema therapist and the possible identification of lymphatic drainage will help the patient tremendously [30,32].

### 5.4. Universal Training and Access to Care

In addition to advancements in clinical techniques, significant disparities in training exist between countries and within programs within individual nations. An international survey revealed that 55% of OMFS practitioners hold a single qualification, primarily a dental degree, while 16% of countries mandate dual qualifications, MD and DDS. Furthermore, 29% of countries favor dual-degree training. This variability in educational standards contributes to confusion within the healthcare system. The establishment of universal training standards or a standardized board examination could facilitate the development of a unified global specialty, thereby supporting OMS surgeons worldwide [33,34]. In the United States, surgical training is typically completed through a four- or six-year residency program. Following residency, trainees may pursue additional fellowship training in oncologic and microvascular reconstruction.

Another critical issue concerns the limited global accessibility of OMFS services. The international distribution of OMSs is markedly uneven: among 104 countries with available data, the median workforce density is 0.518 OMS per 100,000 population. Notably, low-income countries report only 0.015 OMS surgeons per 100,000 individuals, in stark contrast to 1.087 per 100,000 in high-income countries. Moreover, twenty-eight countries (26.9%) have two or fewer OMSs in total. This pronounced disparity in access to OMFS care results in delayed treatment and increased risk of adverse patient outcomes.

## 6. Conclusions

Oral and Maxillofacial Surgery is a dynamic surgical specialty that plays a pivotal role in head and neck reconstruction, providing comprehensive rehabilitation to patients with complex craniofacial defects resulting from trauma, oncologic resection, or congenital anomalies. Oral and Maxillofacial Surgeons are at the forefront of using advanced reconstructive techniques and state-of-the-art technologies to optimize rehabilitation and achieve improved functional and aesthetic outcomes.

The broad scope of the specialty improves patient quality of life and facilitates timely functional rehabilitation, thereby reducing overall healthcare costs and alleviating system strain. Furthermore, as healthcare continues to shift away from the fee-for-service model, OMFS is uniquely positioned to advance value-based care by increasing patient safety, improving efficiency, and enhancing effectiveness through multidisciplinary collaboration in head and neck reconstruction.

## Data Availability

The original contributions presented in this study are included in the article. Further inquiries can be directed to the corresponding author.
